# Observations and Reflections on the Disease Produced by the Action of the Canine Virus on the Human Body

**Published:** 1819-12

**Authors:** James Mease

**Affiliations:** Philadelphia.


FOR THE LONDON MEDICAL AND PHYSICAL JOURNAL.

### *Observations and Reflections on the Disease produced by the Action of the Canine Virus on the Human Body.* By JAMES MEASE, M.D. Philadelphia.

[*Concluded from p.* 378.]

IN preference to caustics, I have always advised the plan first suggested by Dr. HAYGARTH, of Chester, England, viz. the long-continued stream of water on the wound, from the mouth of a tea-kettle: a powerful argument in favour of which remedy is, that, as the poison exists in a watery form, we should expect that water would be its most proper solvent. I have also advised keeping the wound open for some time after, as an additional security. If the wound be small, it ought to be dilated. Here again, however, I think proper to repeat the most judicious advice of Mr. Gilman, which is, "if the knife should enter the wound made by the dog's teeth, to re-commence the operation with a clean knife; for, if we continue to use the knife which is contaminated in consequence of its entering the wounded parts, the operation may be rendered useless, by the sound parts becoming inoculated with the canine virus."—*Gilman on the Bite of a Rabid Animal;* London, 1812.

An inattention to this excellent advice explains the cause of the failure of attempts to extirpate the bitten parts, which sometimes occur.

5. They add to many facts on record, to prove that the interval between the insertion of the virus and the attack of the disease, is not in the least influenced by the distance of the wound from the throat, as formerly and very generally supposed.

6. They show that my objections to the name hydrophobia, or "dread of water," are well-founded.* The symptom in any disease which entitles it to give name thereto, ought always to be present. Now, I have shown that the dread of water, even when it does occur, is only symptomatic of the difficulty of swallowing; and the cases in Richmond, that of the boy Yorke, and many more, prove that there is no instinctive dread of water, and that the symptom is often absent. Some of the patients eagerly desired it to quench their thirst, tried to drink it, and even sometimes succeeded in the attempt. The negro Burwell agreed to take wine a few minutes before he expired, desired them to "be quick, for he was dying:" and he did die shortly after.

The strangury that followed the use of the number of, and extensive, blisters, in the case of the negro Burwell, have destroyed my hopes respecting the good effects which I some years since was led to believe would result from the occurrence of that affection in the disease under consideration. I was induced to think highly of the means to produce that symptom, from its having effected immediate relief in a case of tetanus under the care of Dr. Samuel Brown, of Kentucky; and, from the strong analogy between tetanus and hydrophobia, the influence of that great principle in medicine,—that one irritation often subdues another, the salutary influence of strangury in other diseases,† and the successful use of cantharides for the cure of this disease in Europe,‡ I urged the trial of them therein. I was surely safe in doing so; for, the disease having never been at that time cured by any remedy or any practice, I considered the way open for any new remedy. But I do not think it worthy of confidence now, after its failure to alleviate the symptoms in Burwell's case.

8. The application of caustics, as recommended by Dr. Richard Pearson, of London,§ was vigorously tried by Dr. Trenk, in the treatment of Burwell, without the least good effect, and therefore ought not again to be used. They are painful in the extreme; and, by trusting to them, we may lose time that might be employed with greater chance of success.

---

* The reader will perceive that I still adhere to the opinion of the impropriety of the name *hydrophobia*, notwithstanding the criticisms on my objections to it by Dr. Ferriar, of Manchester. See his *Med. Histories* and *Reflections*.

† Rush's Works, vol. iv. p. 35.

‡ *Diss. de Hydroph.* auct. G. Uiberlacher, of Vienna; C. T. Schwarts *de Hydroph.* Halle, 1813.

§ *Arguments in favour of an Inflam. Diath. in Hydrop. considered;* London, 1798. See also *Med. Rep. N. York*, v. p. 78.

9. The cases in Richmond will for ever, I hope, destroy all confidence in that most shameful imposition the snake-stone, which, with regret I say, the people of Virginia have long been the dupes. If they trust to it hereafter, they will trifle with human life. Why should the public authorities, whose duty it is to remove every evil threatening the safety, health, or lives, of the citizens, permit the chance to be continued of any life being lost, by allowing the possibility of access to so ridiculous a remedy. Grand-juries have more than once presented existing nuisances, and complained of evils likely to injure the public health, of far less importance than that to which I allude. I first heard of the snake-stone by seeing a proposition in the *Richmond Inquirer*, in the autumn of 1805, or beginning of 1806, from J. R. Micou, the proprietor, to sell it for two thousand dollars; the stone to be deposited in Tappahannock, as a central spot, for the use of the subscribing counties; and was so fully convinced that the subscribers to the fund would not only throw away their money, but risk their lives, by trusting to a remedy which had neither well-grounded theory, sure experience, nor common reason, to recommend it, that I felt it my duty to express my disbelief of the virtues attributed to it, and to point out the causes that led to the ill-founded reputation of the stone in question, and to other nostrums with which the world has at various times been duped.* My advice, however, I believe, was not taken. At all events, the reputation of the stone continued undiminished; and my denunciation of it even called forth a writer, who warmly defended its preservative powers against the canine venom. I presume, however, from its twice having recently failed to prevent the disease, although applied under circumstances very favourable to the success of its virtues, (if it is possessed of any,) that his faith, and that of every other person, must be done away with respect to it; and that no one will be so fool-hardy as to trust to it, in the event of the occurrence, in future, of any venomous bite.

The notion of the preservative power of the snake-stone, originated in a district of country (Southern Asia) notorious for the prevalence of superstition of every kind, the deplorable depression of the human mind, and prevalence of jugglers and

* My letter was published in the *Richmond Enquirer*, in April 1806, and republished in Philadelphia, shortly after, in Poulson's paper. I ascribed the deception that takes place in the case of the use of preventives, and the unfounded reputation they enjoy, to the following cases:

1. The absence of madness in the dog that gave the bite,
2. The part bitten being covered. In this case the poisonous saliva is wiped off, and the clear tooth only enters the flesh,
3. The inaptitude of the body to take the disease: the average being not more than one in twenty of persons bitten by dogs actually mad, who are attacked.
4. The careful concealment of many failures of the remedy, and the careful publication of cases of supposed success of prevention.

impostors in religion, in medicine, and the common affairs of life; and it is wonderful how the stone ever obtained the least credit in the United States, the people of which are certainly more free from the influence of superstition, and less liable to be imposed on by pretenders, than those of most countries. Credit, however, it did obtain; no doubt from its having been applied to bites, in which any stone would have answered equally as well as the snake-stone, without any disease following; and the consequence has been the probable sacrifice of two lives! The experience of its inutility has indeed been dearly bought; but the high price of the purchase, as in every transaction of human life, will cause a greater value to be set upon the possession of it.

The remark I make with respect to the unfounded reputation of the snake-stone, in the prevention of the operation of the canine virus on the human system, will apply to all other preventive remedies, with which Europe, Asia, and America, have long abounded.

From Asia we have, first, the tonquin remedy, composed of musk and cinnabar; secondly, the snake-stone. In England, the Ormskirk nostrum, the basis of which was chalk and elecampane root; and, second, the ash-coloured liverwort and black pepper, long held their ground: this last was highly praised by Dr. Mead, the medical oracle of his day; and, upon his authority, it was introduced into the British Dispensatories. In Germany, filings of copper were much celebrated; so also was the *anagallis arvensis,* red pimpernel or chickweed, which was even protected from destruction by law in Switzerland and other governments. In the United States preventive remedies have also multiplied. In Pennsylvania, we have had the anagallis; and Mr. Stoy, of Dauphin county, and Mr. Kettering, of Lancaster county, have been long celebrated for their "uniform success" with it. It failed, however, in two cases that we know of, in which it had a fair trial as a preventive: one to which I allude is that of the late Mr. Huber, of Lancaster.* But Dr. Stoy's widow still keeps up the farce of advertising her nostrum,

---

* See Barton's *Med. and Physical Journal,* vol. ii. p. 125. Dr. Muhlenberg informed me of another case in which it had failed. It also failed in Philadelphia in 1802: see *Med. Repos. N. York,* Hexade 2d, vol. i. p. 105. These three cases are enough to fix its character.

This is the third time I have come forward to deny the virtues of the snake-stone. The second time was in the *Medical Museum* of Philadelphia, vol. v. p. 1; in answer to a publication by the Rev. Wm. H. Harding, in the *Med. Repos.* of New-York, Hexade 2d, vol. v. p. 248, greatly extolling a stone in the possession of the Rev. Lewis Chaustien, of Frederick county, Virginia, which he called the *Chinese* snake-stone. M. C. asserted, that he *had cured about eighty persons in less than two years, some of whom had the hydrophobia on them at the time of application!!* Thus, then, it appears that there are more snake-stones than one in Virginia. I have also one, which was brought from Calcutta a few years since. Others are in the possession of different persons in the United States.

which the late Rev. Dr. Muhlenberg, of Lancaster, the celebrated botanist, who reposed great confidence in it, assured me was composed chiefly of the anagallis.

In New-York we have had the famous remedy of J. M. Crous, for which the legislature gave him a large sum, and which was composed of the powder of a dog's jawbone burnt, the powder of the false tongue of a newly-foaled calf, and verdigris off a copper of George II.* 2. The plant called the scull-cap, or *scutellaria lateriflora*, on the preservative powers of which many still place the most implicit confidence.

Lately, the *alisma plantago*, marsh or water plaintain, comes recommended to us, upon the authority of a Russian peasant, who is said to have long employed it with success, and on that of a Russian counsellor of state, who has written a memoir on the subject. Considering the anagallis as out of the question, I would observe, with respect to the two last, that upon neither of them would I rely, to the exclusion of the local treatment I recommend; as they are all liable to the same objections I have urged against the supposed infallibility of preventives in general.†

* I have given the prescription at length in *Cox's Med. Museum*, vol. v.
† *American Medical Recorder*, vol. ii.

